# Efficacy of Upadacitinib Induction Treatment in Moderate-to-Severe Ulcerative Colitis Including Intestinal Ultrasound Assessment: A Multicenter, Real-World Observational Study

**DOI:** 10.3390/jcm14051695

**Published:** 2025-03-03

**Authors:** Magdalena Kaniewska, Konrad Lewandowski, Michał Krogulecki, Aleksandra Filipiuk, Maciej Gonciarz, Anna Pietrzak, Maria Janiak, Krystian Adrych, Agnieszka Klufczynska, Grażyna Piotrowicz, Maria Kopertowska-Majchrzak, Anatol Panasiuk, Dagmara Mahadea, Piotr Eder, Agnieszka Tarasiuk, Mariusz Rosołowski, Renata Talar-Wojnarowska, Ewa Małecka-Wojciesko, Ariel Liebert, Maria Kłopocka, Ewa Walecka-Kapica, Anita Gąsiorowska, Beata Galińska, Konrad Leśniakowski, Małgorzata Zwolińska-Wcisło, Anna Naumowicz, Jarosław Daniluk, Grażyna Rydzewska

**Affiliations:** 1 Department of Gastroenterology and Internal Medicine, National Medical Institute of the Ministry of the Inferior and Administration, 02-507 Warsaw, Poland; dr.k.lewandowski@icloud.com (K.L.); grazka3558@yahoo.pl (G.R.); 2 Department of Gastroenterology, Military Institute of Medicine, 04-141 Warsaw, Poland; mkrogulecki@gmail.com (M.K.); alfilipiuk@gmail.com (A.F.); m_gonciarz@poczta.fm (M.G.); 3 II Gastroenterology Department, Centre of Postgraduate Medical Education, 00-416 Warsaw, Poland; anpietrzak@gmail.com; 4 Department of Gastroenterology and Hepatology, Medical University of Gdansk, 80-210 Gdansk, Poland; maria.janiak@gumed.edu.pl (M.J.); krystian.adrych@gumed.edu.pl (K.A.); 5 Clinical Hospital of the Ministry of the Interior and Administration, 80-952 Gdansk, Poland; agnieszka.klufczynska@op.pl (A.K.); piotrowicz.grazyna@interia.eu (G.P.); 6 Department of Internal Diseases, General Hospital, 64-400 Miedzychod, Poland; mkopertowskamajchrzak@gmail.com; 7 Department of Internal Diseases and Gastroenterology, Provincial Welded Hospital in Bialystok, 15-950 Bialystok, Poland; anatol@panasiuk.pl; 8 Department of Clinical Medicine, Medical University of Bialystok, 15-254 Bialystok, Poland; 9 Department of Gastroenterology, Dietetics, and Internal Diseases, Poznan University of Medical Sciences, University Clinical Hospital, 61-701 Poznan, Poland; dagmaramahadea@gmail.com (D.M.); piotreder@ump.edu.pl (P.E.); 10 Department of Hypertension, Gastroenterology, and Internal Medicine, Medical University of Bialystok Clinical Hospital, 15-089 Bialystok, Poland; agnieszka.tar7@wp.pl (A.T.); mariusz.rosolowski@umb.edu (M.R.); 11 Department of Internal Medicine and Hypertension, Medical University of Bialystok, 15-089 Bialystok, Poland; 12 Department of Digestive Tract Diseases, Medical University of Lodz, 90-419 Lodz, Poland; r-wojnarowska@wp.pl (R.T.-W.); ewa.malecka-panas@umed.lodz.pl (E.M.-W.); 13 Department of Gastroenterology and Nutritional Disorders, Collegium Medicum in Bydgoszcz, Nicolaus Copernicus University in Torun, 87-100 Bydgoszcz, Poland; ariell1987@wp.pl (A.L.); mariaklopocka@wp.pl (M.K.); 14 Department of Gastroenterology, Medical University of Lodz, Pomorska 251, 92-213 Lodz, Poland; ewawk@op.pl (E.W.-K.); anita.gasiorowska@umed.lodz.pl (A.G.); 15 Department of Gastroenterology and Hepatology, J. Gromkowski Provincial Hospital, 51-149 Wroclaw, Poland; bgalinska@szpital.wroc.pl (B.G.); klesniakowski@szpital.wroc.pl (K.L.); 16 Department of Gastroenterology and Hepatology, Jagiellonian University Medical College, 31-008 Krakow, Poland; mzwcislo@su.krakow.pl; 17 Department of Gastroenterology and Internal Medicine, Medical University of Bialystok, 15-089 Bialystok, Poland; anna.naumowicz@uskwb.pl (A.N.); jaroslaw.daniluk@umb.edu.pl (J.D.); 18 Collegium Medicum, Jan Kochanowski University, 25-369 Kielce, Poland

**Keywords:** upadacitinib, intestinal ultrasound, small molecules, ulcerative colitis, inflammatory bowel disease

## Abstract

**Background:** Upadacitinib (UPA) is a new oral selective Janus Kinase (JAK) inhibitor that has shown high efficacy in the treatment of ulcerative colitis (UC). We present data from a multicenter real-world study. **Methods:** To assess efficacy of UPA, Total Mayo Score (TMS), fecal calprotectin (FC), endoscopy, and intestinal ultrasonography (IUS) were performed. **Results:** The study population included 76 patients. An amount of 26.3% of the patients were biologics and small molecules-naive, while 73.7% were exposed. By Week 8, 93.4% of the patients had achieved a clinical response (94.7% naive vs. 92.9% exposed), 72.4% achieved endoscopic improvement (78.9% vs. 71.4%), and 57.9% had clinical remission (78.9% vs. 51.8%). Endoscopic remission was achieved in 31.6% of patients (35.0% vs. 30.4%) and biochemical remission in 82.1% (53.3% vs. 68.3%). All of the results were not significantly different apart from the steroid-free clinical remission—36.8% (68% vs. 26.8%, *p* = 0.002) after 8 weeks of follow-up. IUS was performed in 33 patients. Bowel wall thickness (BWT), inflammatory fat (iFAT), color Doppler signal (CDS), loss of bowel wall stratification (BWS), and Milano Ultrasound Criteria (MUC) had decreased significantly by Weeks 4 and 8 (*p* < 0.005 for all). Correlation between the IUS results and TMS, FC and endoscopic remission in Week 8 was confirmed (*p* < 0.001). UPA was well tolerated, and no new safety signals were registered in our group. **Conclusions:** In this study, UPA was confirmed to be safe and highly effective in inducing remission in UC patients in both the naive group and the biologically exposed patients. The correlation between the IUS results and TMS, FC, and endoscopic remission provides valuable information for clinicians.

## 1. Introduction

For the past few years, we have been witnessing a revolution in the treatment of inflammatory bowel disease (IBD), mainly due to the emergence of small molecules [1,2,3]. This group of drugs includes Janus kinase (JAK) inhibitors, whose representative is tofacitinib (TOFA), registered in 2018 for the treatment of adult patients with moderate to severe forms of ulcerative colitis (UC) [4].

After several years of experience with small molecules, their place in IBD therapy is well established, owing to their high efficacy and good safety profile, in addition to the convenience associated with the oral form of administration, the lack of immunogenicity, and its rapid onset of action [5,6]. There is some evidence from real-world studies that ultimately established this position, confirming TOFA’s efficacy in UC patients after exposure to anti-tumor necrosis factor-α (anti-TNF-α) therapy [7,8]. Another strength of TOFA, related to its mechanism of action—namely, blocking JAK1 and JAK3—is that it reduces the production of pro-inflammatory cytokines. Thus, TOFA applicability in other inflammatory diseases, such as psoriatic arthritis or plaque psoriasis, is an important aspect of today’s therapy in the context of co-morbidity and extra-intestinal manifestations (EIM) [4,7,8,9,10].

In the new era of the SPIRIT (Selecting End PoInts foR Disease-ModIfication Trials) consensus, where the overarching goal of treatment is to modify the course of the disease in order to improve quality of life, among other things, UPA appears to be an interesting and promising therapeutic avenue [11]. A better understanding of the molecular mechanism made it possible to find a target based on selective action against JAK: higher efficacy (blocking pro-inflammatory JAK1) and safety (bypassing JAK2). This is how the second-generation JAK inhibitors were developed to feature even higher efficacy while having a better safety profile [12].

UPA, registered in March 2022 for the treatment of moderate to severe UC, has an approximately 40-fold higher affinity for JAK1 than JAK2, an approximately 130-fold higher affinity for JAK1 than JAK3, and an approximately 190-fold higher affinity for JAK1 than TYK 2. Phase III UPA trials in patients with moderate to severe active UC and Crohn’s disease (CD) have demonstrated its efficacy in achieving and maintaining clinical and endoscopic remission not only in anti-TNF-exposed patients but also in those after TOFA exposure [13,14].

The purpose of our study was to evaluate the efficacy and safety of 45 mg of UPA daily in inducing remission in patients with moderate to severe ulcerative colitis (UC). The primary disease activity parameters included in the primary and secondary endpoints were evaluated to assess the drug’s efficacy. The assessment was performed multidimensionally—clinically, biochemically, endoscopically, and using an ultrasound in a real-world setting.

## 2. Materials and Methods

### 2.1. Patients

We conducted a study in 15 centers nationwide in which we included 76 patients with a confirmed diagnosis of UC. The patients were at least 18 years old and had received UPA for at least 8 weeks between June 2023 and January 2024. Patients had moderate to severe UC flare (Total Mayo score > 6 points) with inadequate response, loss of response, or intolerance to corticosteroids and immunosuppressants. Patients were allowed to be treated with biological or small molecules therapy (except upadacitinib) in the past. Key exclusion criteria included a diagnosis of Crohn’s disease, indeterminate colitis, toxic megacolon, active infection, and treatment with upadacitinib in the past.

### 2.2. Bioethics Committee Approval

The study protocol was approved by the Bioethics Committee of the National Medical Institute of the Ministry of the Inferior and Administration (approval number 47/2024, date: 4 June 2024).

### 2.3. Treatment

Patients were treated with upadacitinib 45 mg daily during 8 weeks of induction period.

### 2.4. Assessment

The primary endpoints were clinical response (defined as a reduction ≥ 3 points in Total Mayo Score), endoscopic improvement (defined as endoscopic subscore ≤ 1 without friability), and clinical remission (defined as ≤2 points in Total Mayo Score with or without no individual subscore > 1). As secondary endpoints, we distinguished steroid-free clinical remission (clinical remission without using corticosteroids), biochemical remission (fecal calprotectin level below 250 μg/g), and endoscopic remission (defined as endoscopic score of 0). Also, we evaluated the safety profile according to recorded treatment-related adverse events and serious adverse events. We also present our data in terms of the achievement of primary and secondary endpoints in the whole group, as in a subanalysis of subgroups: patients naive to biologic and JAK treatment (naive) and after exposure to these drugs (exposed).

### 2.5. Intestinal Ultrasound

In addition, the intestinal ultrasound (IUS) was carried out at two centers as the standard of care, so we present the results of 33 patients in the context of parameters for ultrasound evaluation of the bowel. We aimed to evaluate, in a sub-comparison with IUS, the usefulness of this tool in terms of assessing response to treatment. Thus, the primary endpoints of this sub-analysis were an assessment of disease activity in terms of the following parameters within each bowel segment: bowel wall thickness (BWT), inflammatory fat (iFAT), color Doppler signal (CDS), loss of bowel wall stratification (BWS), and Milano Ultrasound Criteria (MUC) [15,16]. Because secondary endpoints were used to evaluate the usefulness of IUS for assessing disease activity, correlation between MUC and Total Mayo Score, fecal calprotectin (FC), and endoscopic remission could be examined.

### 2.6. Study Design

During patient visits in Weeks 0, 4, and 8, laboratory tests, Total Mayo Score, FC, endoscopy, and adverse effects and serious adverse events were evaluated. In addition, 33 patients at two independent centers had IUS performed at each of the three visits (Week 0, 4, 8).

### 2.7. Statistical Analysis

The software program R version 4.1.2 was employed for the analysis. The total study group consisted of 76 patients. For the description of the numeric variables, mean and SD or median and IQR were used, depending on the normality of the distribution. Categorical variables are presented with counts and percentages of the respective group. Comparisons between visits were performed on paired observations using a paired *t*-test, Wilcoxon test, McNemar test, or Friedman test, as appropriate. Spearman’s correlation analysis was used to verify relationships between MUC and fecal calprotectin or between MUC and Total Mayo Score. As endoscopic remission, defined as MAYO 0 or I endoscopic image, is a categorical parameter (not quantitative); correlation with the correlation coefficient cannot be assessed. The predictive quality of MUC < 6.2 was assessed by calculating sensitivity, specificity, positive predictive value, negative predictive value, and accuracy. All statistical calculations assumed *α* = 0.05.

## 3. Results

### 3.1. Total Group

The group that was followed-up with for 8 weeks consisted of 76 patients, of whom 35.5% were female. The mean age was 37.18 ± 11.63 years, ranging from 20 to 73 years. The mean BMI was 23.00 ± 3.52 kg/m^2^. The median time from diagnosis was 5 years. An amount of 55.3% had extensive UC (Montreal E3), 39.5% had left-sided UC (E2), and 5.2% had proctitis (E1). An amount of 74.7% were biologic-/small molecules-exposed patients and 25.3% were naive patients. An amount of 26.7% had been exposed to two drugs, 17.3% to three drugs, 18.7% to one drug, 10.7% to four drugs, and 1.3% to five (infliximab, vedolizumab, TOFA, ustekinumab, mirikizumab). An amount of 30.2% (23) had been exposed to infliximab. The details of treatment history, laboratory results, baseline disease activity, and comorbidities are shown in Table 1.

At Week 4, data were available for 44 patients: 90.9% (40) of patients receiving UPA achieved a clinical response, 56.8% (25) achieved endoscopic improvement, and 59.1% (26) clinical remission. Endoscopic remission was obtained in 38.6% (17), steroid-free clinical remission in 27.3% (12), and biochemical remission in 56.8% (25) after 4 weeks of follow-up.

At Week 8, data were available for all 76 patients: 93.4% (71) of patients receiving UPA achieved a clinical response, 72.4% (55) endoscopic improvement, and 57.9% (44) clinical remission. Endoscopic remission was obtained in 31.6% (24), steroid-free clinical remission in 36.8% (26), and biochemical remission in 82.1% (36) after 8 weeks of follow-up.

### 3.2. Intestinal Ultrasound (IUS) Group

The IUS group that was followed-up with for 8 weeks consisted of 33 patients, of whom 60.6% had extensive UC (E3), 36.4% had left-sided UC (E2), and one had proctitis (E1). The MUC for the worst segment was 9.65 ± 1.66 on average (data showing the activity of UC are included in Appendix A Table A1), while IUS data at Week 0 are in Table 2.

At Weeks 4 and 8, BWT, iFat, CDS, and BWS had decreased significantly in all segments (except for the terminal ileum) (*p* < 0.05; Table 3). The MUC was also significantly lower at Week 4 (MD = −2.92 CI_95_ [−3.92; −1.92], *p* < 0.001) and Week 8 (MD = −4.18 CI_95_ [−5.09; −3.28], *p* < 0.001; (Table 3).

At defined intervals, we tested the usefulness of MUC < 6.2 as a diagnostic test for clinical remission expressed in Total Mayo Score, for FC below 250 μg/g, and for endoscopic remission. At Week 4, MUC < 6.2 as a diagnostic test for clinical remission expressed in Total Mayo Score had a sensitivity of 54.55% and a specificity of 77.27%; for FC below 250 μg/g, it had a sensitivity of 42.11% and a specificity of 75.00%; and for endoscopic remission, it had a sensitivity of 50.00% and a specificity of 70.37%.

At Week 8, MUC < 6.2 had a sensitivity of 83.33% and a specificity of 66.67% for clinical remission expressed in Total Mayo Score, a sensitivity of 74.07% and a specificity of 100.00% for FC below 250 μg/g, and a sensitivity of 90.00% and a specificity of 52.17% for endoscopic remission (Appendix A Table A2).

**Figure 1:**
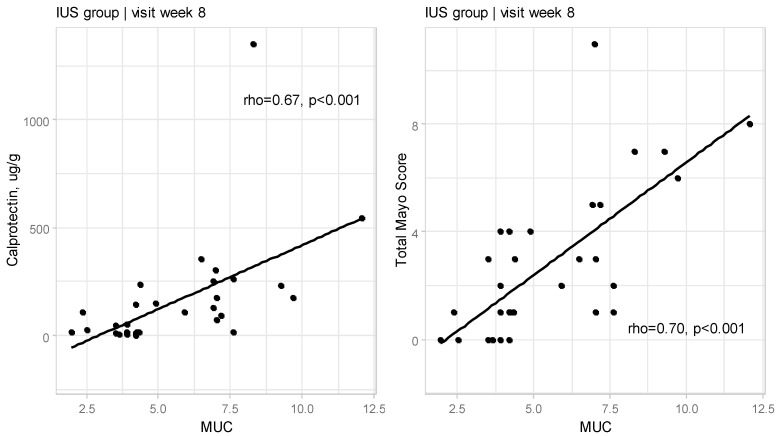
Relationships between Milano Ultrasound Criteria and calprotectin as well as Total Mayo Score at Week 8 for IUS group (rho—Spearman’s correlation coefficient).

At Week 8, a significant correlation between MUC and FC (rho = 0.67, *p* < 0.001) and between MUC and Total Mayo Score (rho = 0.70, *p* < 0.001; Figure 1) was confirmed.

**Figure 2:**
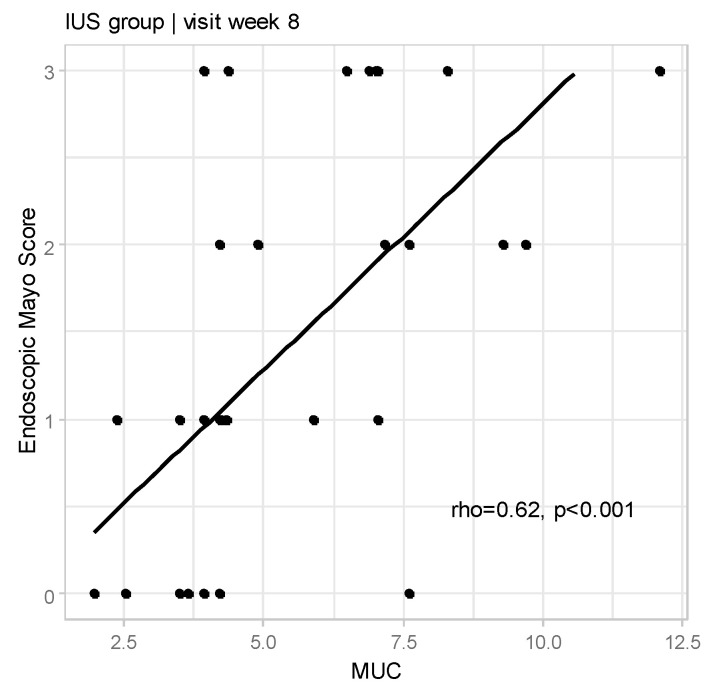
Relationships between Milano Ultrasound Criteria and Endoscopic Mayo Score at Week 8 for IUS group (rho—Spearman’s correlation coefficient).

We also confirmed the correlation between MUC and Endoscopic Mayo Score (rho = 0.62, *p* < 0.001; Figure 2).

**Figure A1:**
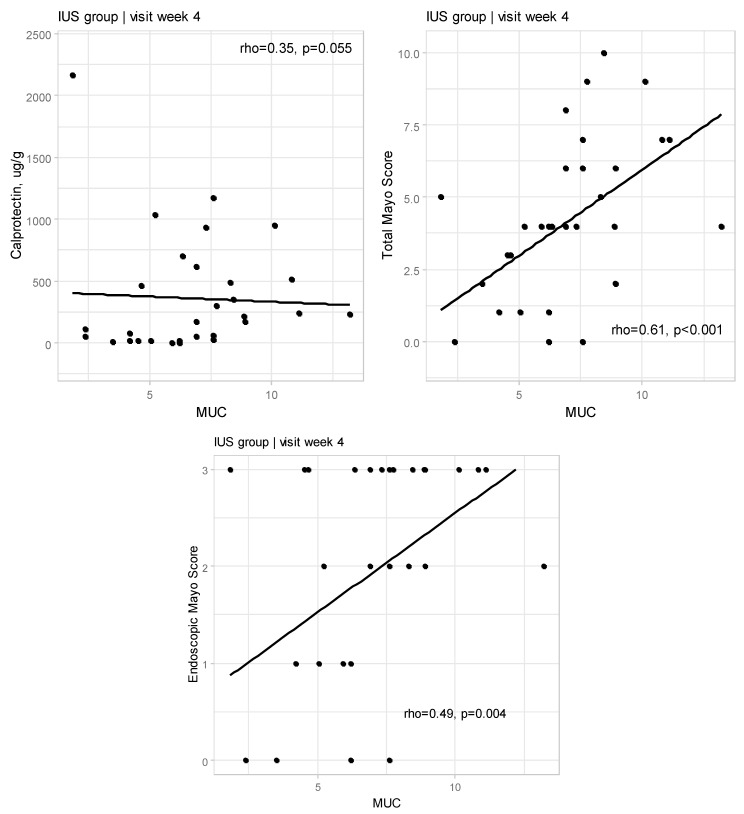
Relationships between Milano Ultrasound Criteria and calprotectin as well as Total Mayo Score and with endoscopic Mayo Score at Weeks 4 for IUS group (rho—Spearman’s correlation coefficient).

We also estimated the correlation between MUC and Total Mayo Score and Endoscopic Mayo score in Week 4, which was confirmed but the correlation between MUC and FC was not statistically significant (*p* = 0.055) (Appendix A Figure A1).

### 3.3. Safety Profile

At Week 4, some AEs were reported: hyperlipidemia in 8 cases, hypertriglyceridemia in 4 cases, mild leukopenia in 4 cases, mild anemia in 2 cases, and 1 Herpes simplex *virus* infection. We observed one serious adverse event (SAE)—catheter-related sepsis on intravenous nutrition—but it did not require the suspension or termination of treatment. At Week 8, some AEs were noted: hyperlipidemia in 13 cases, hypertriglyceridemia in 6 cases, mild leukopenia in 4 cases, mild anemia in 2 cases, and 1 case of mild neutropenia. There were no SAEs at this time-point. All the above data are presented in Table 4.

### 3.4. Efficacy of Upadacitinib in the Naive and Biologically and Small Molecules-Exposed Patients

At Week 8, a clinical response was achieved in 94.7% of the naive patients, 92.9% of the exposed (*p* > 0.999), endoscopic improvement was obtained in 78.9% of the naive patients, 71.4% of the exposed (*p* = 0.652), and 78.9% of naive patients and 71.4% of exposed (*p* = 0.053) achieved clinical remission. Endoscopic remission was confirmed in 35.0% of naive patients, 30.4% of exposed (*p* = 0.918), and steroid-free clinical remission was achieved by 68.0% of naive patients (*p* = 0.002) and 26.8% of exposed. A reduction in FC to values below 250 μg/g, defined as biochemical remission, was found in 53.3% naive patients and 68.3% of exposed (*p* = 0.448) (All data are shown in Appendix A Table A3).

## 4. Discussion

This is the first study to confirm the efficacy of UPA using the simultaneous assessment of the intestinal ultrasound, not only in the naive patient group but also in the biologically exposed patient group. Thus, bearing in mind the results at Week 8, such as clinical response in 93.4%, endoscopic improvement in 72.4%, clinical remission in 57.9%, endoscopic remission in 31.6%, steroid-free clinical remission in 36.8%, and biochemical remission in 82.1%, UPA seems to be highly effective in inducing remission in patients with moderate-to-severe UC patients.

Our results significantly exceeded the rates from the UPA clinical trials in UC (U-ACHIEVE, U-ACCOMPLISH), which may be related to several aspects: differences in endpoint definitions, and different, more difficult group included in the phase III clinical trials. In both UPA clinical trials, clinical remission was defined very strictly (stool frequency subscore of 1 and no more than at baseline, rectal bleeding subscore of 0, and endoscopic subscore of 1 without tenderness); this endpoint was achieved in 26% and 33.5% of patients [17]. Of note, however, is the fact that in both clinical trials, more than half of the patients were naive. It should be remembered that the inclusion criteria were also very restrictive, including the imposition of a period of stable therapy before treatment in order to avoid errors resulting from the combination of therapies. In our group, however, this would have been completely impossible, as these were mostly patients who had already undergone ineffective therapies and required rapid action to improve their condition. Despite the lack of such observations, we cannot rule out a short-term effect of the previous therapy due to the absence of a wash-out phase. However, this hypothesis is clearly contradicted by the safety profile, which should be assessed as good.

The power of our results does not flow from the percentage of endpoints obtained alone, but more from the sub-analysis, such as the evaluation of efficacy in various groups of patients: naive, exposed, IFX-exposed, and after the failure of four advanced therapies. At the moment, data on the efficacy of UPA in patients after IFX exposure are very encouraging, but data on patients with multidrug-resistant disease are still lacking. In our study, we decided to include eight patients after IFX exposure (anti-TNF failers). Clinical remission was achieved in 62.5% of them (5), while endoscopic remission was achieved in 75% (6), which may indicate the efficacy of UPA in such a group of patients. However, we are mindful of the fact that this group is small, and these results should be addressed critically.

To assess the speed of action in our patients, we evaluated them after 4 weeks, observing a clinical response in 90.9%, clinical remission in 59.1%, and endoscopic remission in 22.7%, which is impressive. This rapid onset made it possible to discontinue steroids and achieve high steroid-free clinical remission in 27.3%, thus reducing their side effects.

UPA also has a good safety profile, and the most common AE was hyperlipidemia. AEs were found in 26.3% of patients at Week 4 and in 34.2% at Week 8; however, they did not require a discontinuation of therapy. There was one SAE in our study that was most likely not related to the drug: catheter-related sepsis on intravenous nutrition. The use of UPA was not stopped, and after treatment with antibiotics and removal of the catheter, the symptoms were resolved.

In a prospective study by Friedberg et al., the effectiveness of UPA in inducing remission in patients with UC and CD was investigated. In a group of 44 patients with UC, clinical remission was observed after 8 weeks in 81.5% and clinical response in 85.2% [18]. These results are much better than ours, but the group was half the size. It should be noted that—in contrast to our group—this was a single-center study. Similarly, to our study, a group of researchers from Chicago assessed some patients after exposure to several drugs, but, ultimately, they did not present an analysis of the treatment effects of this subgroup. What remains surprising is the completely different AE profiles for patients with UC and Crohn’s disease (CD), with acne dominating in 32.4% [18]. No one in our group experienced acne during therapy; the lack of such data could be related to the fact that patients used local treatment on their own, not linking it to the effect of the drug. In our study, we also assessed the lipid profile, which was not the case in the Friedberg study, so it is not possible to compare the incidence of hyperlipidemia (which was the most common in our patients).

Dalal et al. also retrospectively studied the effectiveness of UPA in 76 patients with UC, among which 91% had prior anti-TNF exposure. This was a two-center study, but the authors did not evaluate clinical remission. Steroid-free clinical remission was achieved by almost twice as many patients as in our study (64.0% vs. 36.8%) [19]. The clinical response and endoscopic remission was very similar to that in our study, amounting to 84.2% and 31.6%. They also assessed the safety profile as good but did not assess the lipid profile.

Berinstein et al. studied effectiveness of UPA in patients with acute severe UC (ASUC). It was a multicenter study with 25 patients with ASUC treated with upadacitinib. The primary outcome was 90-day colectomy rate. An amount of 24% patients underwent colectomy, 83% of those, who did not undergo colectomy experienced steroid-free clinical remission. In our study we did not have any serious adverse events ended with colectomy [20].

Another RWA data from Boneschansker L. that included 119 patients treated with tofacitinib and 35 patients treated with upadacitinib. Most of them had previously used anti-tumor necrosis factor therapy (97%), approximately 70% in both groups had failed vedolizumab treatment. After induction treatment, 40% of patients treated with UPA achieved clinical remission compared with TOFA (18%; *p* = 0.006) [21]. In our study, we did not compare patients treated with UPA to TOFA, but we achieved clinical remission in almost 58% of patients.

The great advantage of our study is the assessment of the response to UPA treatment using IUS. We observed a significant decrease in disease activity parameters as assessed by BWT, iFat, CDS, and BWS. Their significant decrease was noticeable after 4 weeks and this effect persisted and even strengthened in the 8th week. It is also very important to assess the MUC coefficient, which not only dropped significantly in just 4 weeks, but by Week 8 had decreased by almost half. At each stage of treatment, we assessed the sensitivity and specificity of MUC and its correlation with Total Mayo Score and FC concentration. We demonstrate that MUC is a better marker for the Total Mayo Score, which we attribute primarily to the better accuracy of this scale in assessing the severity of the disease. However, in the context of disease activity, it is the sensitivity and specificity of MUC in relation to endoscopic remission that is most important, which is particularly evident at Week 8. However, it is noteworthy that, especially in Week 8, the sensitivity and specificity of MUC on endoscopic assessment were 90.00% and 52.17%, respectively, which means that this parameter reflects well the severity of inflammatory changes on endoscopic examination.

De Voogd et al. performed a prospective IUS evaluation in 51 UC patients starting treatment with IFX, TOFA, or vedolizumab (VDZ). Clinical outcomes, biochemical parameters, and IUS were assessed at baseline and Weeks 2 and 6 of therapy. BWT was lower at Week 6 in the patients who achieved endoscopic improvement (3.0 ± 1.2 mm vs. 4.1 ± 1.3 mm; *p* = 0.026), remission (2.5 ± 1.2 mm vs. 1 ± 1.1 mm; *p* = 0.002), and clinical remission (3.01 ± 1.34 mm vs. 3.85 ± 1.20 mm; *p* = 0.035). Additionally, BWT decreased significantly at Week 2 for infliximab and tofacitinib and at Week 6 for vedolizumab [22]. Even though completely different time-points were used in this study, and MUC was calculated, the IUS assessment of the speed of action in particular seems to be a typical feature of JAK. When comparing the results of Week 6 of TOFA therapy with Week 8 of UPA therapy in the context of UPA, they should be considered comparable.

Moreover, in a multicenter Australian study, the effectiveness of UPA in the treatment of severe acute ulcerative colitis was assessed in six patients, also using IUS. All six patients demonstrated a clinical response to UPA induction on hospital admission. Four patients achieved clinical remission without corticosteroids by Week 8, including complete resolution of rectal bleeding and transmural healing assessed by IUS [23]. In our study, there was also a significant improvement in BWT by almost half; a reduction in >25% from the baseline value is defined as a response to treatment [15]. This indicates not only the effectiveness of UPA, but above all the high sensitivity of this method as a non-invasive tool.

The main advantages of this study were its real-world, multicenter nature, the assessment of the effectiveness of therapy not only after IFX but also in patients with multidrug-resistant disease (after four advanced therapies), and the substudy with IUS. The small number of patients in the subanalysis (eight patients in the group exposed to four advanced therapies) is also a limitation, because the conclusions drawn from this small amount of data are limited. The second limitation is the lack of IUS assessment of patients a few days after the start of therapy, which would have better demonstrated the speed of the drug’s effect.

## 5. Conclusions

In this multicenter, real-world study, UPA was proven to be highly effective in the induction of remission in patients with moderate to severe UC. This effectiveness was observed in both the naive patient group and the biologically and small molecules-exposed patients, which makes UPA an interesting possibility for all UC patients. These results were observed by simultaneous assessment of IUS. The correlation between results from IUS and Total Mayo Score, FC, and endoscopic remission was confirmed. IUS is a valuable, non-invasive tool for monitoring UC patients. Another strength of UPA was safety profile, which was associated with a small number of AEs.

## Figures and Tables

**Table 1:** Baseline characteristics in total group.

Variable	UC (*n* = 76)
Age, Y, M ± SD	42.18 ± 11.63
Gender, female, *n* (%)	27 (35.5)
Age at IBD diagnosis, Y, M ± SD	37.18 ± 11.63
BMI, kg/m^2^, M ± SD	23.00 ± 3.52
Duration of IBD, Y, Me (IQR)	5.00 (3.00; 8.00)
Extension	
Pancolitis, *n* (%)	42 (55.3)
Left-sided, *n* (%)	30 (39.5)
Proctitis, *n* (%)	4 (5.2)
Resistant to steroids, *n* (%)	26 (34.2)
Dependent on steroids, *n* (%)	49 (64.5)
Systemic steroids at baseline, *n* (%)	30 (39.5)
Budesonide MMX at baseline, *n* (%)	7 (9.2)
Biologic/small molecule, exposed	
Naive, *n* (%)	20 (26.3)
Exposed, *n* (%)	56 (73.7)
Number of prior advanced therapies	
1, *n* (%)	14 (18.7)
2, *n* (%)	20 (26.7)
3, *n* (%)	13 (17.3)
4, *n* (%)	8 (10.7)
5, *n* (%)	1 (1.3)
Fecal calprotectin, μg/g, Me (IQR)	1000.00 (478.00; 1736.00)
Mayo Endoscopic Score	
0, *n* (%)	0 (0.0)
1, *n* (%)	1 (1.3)
2, *n* (%)	26 (34.2)
3, *n* (%)	49 (64.5)
Total Mayo Score	
Remission, *n* (%)	0 (0.0)
Mild activity, *n* (%)	0 (0.0)
Moderate activity, *n* (%)	47 (61.8)
Severe activity, *n* (%)	29 (38.2)
Medical history	
Cancer, *n* (%)	0 (0.0)
Non-melanoma skin cancer (NMSC), *n* (%)	2 (2.6)
Myocardial infarction/stroke, *n* (%)	0 (0.0)
Venous thromboembolism, *n* (%)	1 (1.3)
Smoking, *n* (%)	4 (5.3)
Drug hypersensitivity, *n* (%)	8 (10.5)

Prior advanced therapies: Inflik.

**Table 2:** Comparison of IUS outcomes in Weeks 4 and 8 vs. Week 0.

**Variable**	**BWT**			**iFat**			**CDS**			**BWS**		
	**Week 0**	**Week 4**	***p***	**Week 0**	**Week 4**	***p***	**Week 0**	**Week 4**	***p***	**Week 0**	**Week 4**	***p***
Rectum	5.05 ± 1.06	3.71 ± 1.42	<0.001	2.00 (1.00; 2.00)	1.00 (0.00; 1.00)	<0.001 ^1^	1.00 (1.00; 1.00)	1.00 (1.00; 1.00)	0.020 ^1^	2.00 (2.00; 3.00)	1.00 (0.00; 1.00)	<0.001 ^1^
Sigmoid	4.66 ± 1.11	3.40 ± 1.43	<0.001	1.70 ± 0.47	0.67 ± 0.78	<0.001	1.00 (1.00; 1.00)	1.00 (0.00; 1.00)	0.002 ^1^	1.94 ± 0.79	0.67 ± 0.74	<0.001
Descending	3.92 ± 1.11	3.07 ± 1.11	<0.001	1.18 ± 0.85	0.45 ± 0.62	<0.001	0.79 ± 0.42	0.48 ± 0.51	0.002	1.09 ± 0.98	0.45 ± 0.62	<0.001
Transverse	3.10 (2.80; 4.90)	2.60 (2.00; 3.00)	<0.001 ^1^	0.79 ± 0.86	0.27 ± 0.45	0.001	0.55 ± 0.51	0.27 ± 0.45	0.005	0.00 (0.00; 1.00)	0.00 (0.00; 0.00)	0.004 ^1^
Ascending	2.90 (2.50; 3.50)	2.70 (2.00; 3.00)	0.005 ^1^	0.58 ± 0.75	0.15 ± 0.36	0.003	0.52 ± 0.51	0.18 ± 0.39	0.001	0.00 (0.00; 1.00)	0.00 (0.00; 0.00)	0.015 ^1^
Cecum	2.80 (2.50; 3.20)	2.50 (2.00; 2.80)	0.006 ^1^	0.00 (0.00; 1.00)	0.00 (0.00; 0.00)	0.008 ^1^	0.30 ± 0.47	0.09 ± 0.29	0.017	0.00 (0.00; 0.00)	0.00 (0.00; 0.00)	0.021 ^1^
Terminal ileum, backwash ileitis	2.21 ± 0.53	2.10 ± 0.52	0.270	0.00 (0.00; 0.00)	0.00 (0.00; 0.00)	-	0.00 (0.00; 0.00)	0.00 (0.00; 0.00)	-	0.00 (0.00; 0.00)	0.00 (0.00; 0.00)	-
**Variable**	**BWT**			**iFat**			**CDS**			**BWS**		
	**Week 0**	**Week 8**	***p***	**Week 0**	**Week 8**	***p***	**Week 0**	**Week 8**	***p***	**Week 0**	**Week 8**	***p***
Rectum	5.05 ± 1.06	3.14 ± 1.22	<0.001	2.00 (1.00; 2.00)	0.00 (0.00; 1.00)	<0.001 ^1^	1.00 (1.00; 1.00)	0.00 (0.00; 1.00)	<0.001 ^1^	2.30 ± 0.68	0.48 ± 0.67	<0.001
Sigmoid	4.66 ± 1.11	3.12 ± 1.16	<0.001	2.00 (1.00; 2.00)	0.00 (0.00; 1.00)	<0.001 ^1^	1.00 (1.00; 1.00)	0.00 (0.00; 1.00)	<0.001 ^1^	2.00 (1.00; 2.00)	0.00 (0.00; 1.00)	<0.001 ^1^
Descending	3.92 ± 1.11	2.84 ± 0.98	<0.001	1.00 (0.00; 2.00)	0.00 (0.00; 0.00)	<0.001 ^1^	0.79 ± 0.42	0.33 ± 0.48	<0.001	1.00 (0.00; 2.00)	0.00 (0.00; 0.00)	<0.001 ^1^
Transverse	3.10 (2.80; 4.90)	2.60 (2.00; 3.00)	<0.001 ^1^	1.00 (0.00; 2.00)	0.00 (0.00; 0.00)	0.001^1^	0.55 ± 0.51	0.18 ± 0.39	0.001	0.00 (0.00; 1.00)	0.00 (0.00; 0.00)	0.002 ^1^
Ascending	2.90 (2.50; 3.50)	2.50 (2.00; 3.00)	<0.001 ^1^	0.58 ± 0.75	0.03 ± 0.17	<0.001	1.00 (0.00; 1.00)	0.00 (0.00; 0.00)	0.001 ^1^	0.00 (0.00; 1.00)	0.00 (0.00; 0.00)	0.008 ^1^
Cecum	2.80 (2.50; 3.20)	2.40 (2.00; 2.50)	0.001 ^1^	0.00 (0.00; 1.00)	0.00 (0.00; 0.00)	0.008 ^1^	0.00 (0.00; 1.00)	0.00 (0.00; 0.00)	0.003 ^1^	0.00 (0.00; 0.00)	0.00 (0.00; 0.00)	0.021 ^1^
Terminal ileum, backwash ileitis	2.21 ± 0.53	2.01 ± 0.42	0.052	0.00 (0.00; 0.00)	0.00 (0.00; 0.00)	-	0.00 (0.00; 0.00)	0.00 (0.00; 0.00)	-	0.00 (0.00; 0.00)	0.00 (0.00; 0.00)	-

Data are presented as mean ± standard deviation or median (interquartile range), depending on normality of distribution. Difference between visits measured with paired *t*-test or Wilcoxon test, ^1^ as appropriate.

**Table 3:** Comparison of disease activity in IUS group at defined intervals, Weeks 0, 4, and 8.

Variable	Week 0	Week 4	MD (95% CI)	*p*	Week 8	MD (95% CI)	*p*
Fecal calprotectin, μg/g, Me (IQR)	681.00 (430.00; 1178.50)	174.00 (19.75; 501.35)	−507.00 (−761.75; −240.00)	<0.001 ^1^	70.00 (16.00; 177.50)	−611.00 (−954.00; −453.75)	<0.001 ^1^
Total Mayo Score							
Remission	0 (0.0)	11 (33.3)	-	<0.001	10 (30.3)	-	<0.001 ^2^
Mild activity	0 (0.0)	12 (36.4)			8 (24.2)		
Moderate activity	20 (60.6)	10 (30.3)			6 (18.2)		
Severe activity	13 (39.4)	0 (0.0)			9 (27.3)		
MUC, M ± SD	9.65 ± 1.66	6.73 ± 2.58	−2.92 (−3.92; −1.92)	<0.001	5.47 ± 2.33	−4.18 (−5.09; −3.28)	<0.001

Difference between visits measured with Wilcoxon test, ^1^ McNemar test, ^2^ or Friedman test, ^2^ as appropriate.

**Table 4:** Characteristics of adverse events and serious adverse events.

Variables	Week 4	Week 8
Neutropenia (Week 0 ≥ 1.8 and follow-up < 1.8), *n*	4	4
Lymphopenia (Week 0 ≥ 0.8 and follow-up < 0.8), *n*	0	1
Anemia (females: Week 0 ≥12 and follow-up < 12; males: Week 0 ≥ 13 and follow-up < 13), *n*	2	2
AST (Week 0 ≤ 100 and follow-up > 100), *n*	0	0
ALT (Week 0 ≤ 100 and follow-up > 100), *n*	0	0
LDL (Week 0 ≤ 135 and follow-up > 135), *n*	8	13
TGL (Week 0 ≤ 150 and follow-up > 150), *n*	4	6
Need for colectomy, *n*	0	0
Infection (HSV and catheter-related sepsis on intravenous nutrition), *n*	2	0
Cancer, *n*	0	0
NMSC, *n*	0	0
Renal failure, *n*	0	0
MACE, *n*	0	0
VTE, *n*	0	0
Creatine kinase, *n*	0	0
* SAE (catheter-related sepsis on intravenous nutrition), *n*	1	0
Any side effect (any of above), *n* (%)	20 (26.3)	26 (34.2)

Data are presented as number of patients for laboratory abnormalities and as number of patients (% of group) for Other and Any side effects (SAE). * Including severe infections, HSV, anemia, neutropenia, and lymphopenia.

## Data Availability

Data are contained within the article.

## Abbreviations

AEs	Adverse events
ALT	Alanine Aminotransferase
anti-TNF-α	Anti-tumor necrosis factor-α
AST	Aspartate aminotransferase
BMI	Body Mass Index
BWS	Loss of bowel wall stratification
BWT	Bowel wall thickness
CD	Crohn’s disease
CDS	Color Doppler signal
CI	Confidence interval
EIM	Extra-intestinal manifestations
FC	Fecal calprotectin
HSV	Herpes simplex virus
IBD	Inflammatory bowel disease
iFAT	Inflammatory fat
IFX	Inflixymab
IUS	Intestinal ultrasonography
IQR	Interquartile range
JAK	Janus Kinase
LDL	Low-Denstity Lipoprotein
M	Mean
MACE	Major adverse cardiac events
MD	Median difference
Me	Median
MUC	Milano Ultrasound Criteria
MMX	Multimatrix
NMSC	Nonmelanoma skin cancer
NPV	Negative predictive value
PPV	Positive predictive value
RWE	Real Word Experience
SAE	Serious adverse event
SD	Standard deviation
TGL	Triglycerides
TMS	Total Mayo score
TOFA	Tofacitinib
UC	Ulcerative colitis
UPA	Upadacitinib
VTE	Venous thromboembolism
Y	Years

## Appendix A

**Table A1:** Baseline characteristics among patients with IUS assessment.

Variable	IUS (*n* = 33)
Duration of IBD, Y, Me (IQR)	4.00 (3.00; 8.00)
Extension	
Pancolitis, *n* (%)	20 (60.6)
Left-sided, *n* (%)	12 (36.4)
Proctitis, *n* (%)	1 (3.0)
Systemic steroids at baseline, *n* (%)	30 (39.5)
Budesonide MMX at baseline, *n* (%)	7 (9.2)
Fecal calprotectin, μg/g, Me (IQR)	860.00 (468.00; 1248.44)
Mayo Endoscopic Score	
0, *n* (%)	0 (0.0)
1, *n* (%)	1 (3.0)
2, *n* (%)	16 (48.5)
3, *n* (%)	16 (48.5)
Total Mayo Score	
Remission, *n* (%)	0 (0.0)
Mild activity, *n* (%)	0 (0.0)
Moderate activity, *n* (%)	23 (69.7)
Severe activity, *n* (%)	10 (30.3)
Milano Ultrasound Criteria, mean ± SD	9.65 ± 1.66

IQR—interquartile range, IUS—intestinal ultrasound, M—mean, Me—median, SD—standard deviation.

**Table A2:** Quality of MUC (for worst segment) lower than 6.2 in predicting Total Mayo Score lower than 3 in predicting fecal calprotectin level lower than 250 µg/g and in predicting endoscopic remission, at Weeks 4 and 8.

**Week 4**	**Total Mayo Score**		**Sensitivity, %**	**Specificity, %**	**PPV, %**	**NPV, %**	**Accuracy, %**
	**< 3**	**≥3**					
MUC < 6.2	6	5	54.55	77.27	54.55	77.27	69.70
MUC ≥ 6.2	5	17					
**Week 4**	**Fecal calprotectin**		**Sensitivity, %**	**Specificity, %**	**PPV, %**	**NPV, %**	**Accuracy, %**
	**<250 µg/g**	**≥250 µg/g**					
MUC < 6.2	8	3	42.11	75.00	72.73	45.00	54.84
MUC ≥ 6.2	11	9					
**Week 4**	**Endoscopic MAYO Score**		**Sensitivity, %**	**Specificity, %**	**PPV, %**	**NPV, %**	**Accuracy, %**
	**0**	**I-III**					
MUC < 6.2	3	8	50.00	70.37	27.27	86.36	66.67
MUC ≥ 6.2	3	19					
**Week 8**	**Total Mayo Score**		**Sensitivity, %**	**Specificity, %**	**PPV, %**	**NPV, %**	**Accuracy, %**
	**<3**	**≥3**					
MUC < 6.2	15	5	83.33	66.67	75.00	76.92	75.76
MUC ≥ 6.2	3	10					
**Week 8**	**Fecal calprotectin**		**Sensitivity, %**	**Specificity, %**	**PPV, %**	**NPV, %**	**Accuracy, %**
	**<250 µg/g**	**≥250 µg/g**					
MUC < 6.2	20	0	74.07	100.00	100.00	46.15	78.79
MUC ≥ 6.2	7	6					
**Week 8**	**Endoscopic MAYO Score**		**Sensitivity, %**	**Specificity, %**	**PPV, %**	**NPV, %**	**Accuracy, %**
	**0**	**I-III**					
MUC < 6.2	9	11	90.00	52.17	45.00	92.31	63.64
MUC ≥ 6.2	1	12					

**Table A3:** Primary and secondary endpoints in subgroups: naïve and exposed after 8 weeks of treatment.

Variable	Naive, *n* = 20	Exposed, *n* = 56	χ2	*p*
Clinical response, %	94.7	92.9	-	>0.9991
Endoscopic improvement, %	78.9	71.4	0.20	0.652
Clinical remission, %	78.9	51.8	3.76	0.053
Endoscopic remission, %	35.0	30.4	0.01	0.918
Steroid-free clinical remission, %	68.0	26.8	9.90	0.002
Biochemical remission, %	53.3	68.3	0.58	0.448
